# Correction: Yu et al. Comparison of Physical and Biochemical Characterizations of SARS-CoV-2 Inactivated by Different Treatments. *Viruses* 2022, *14*, 1938

**DOI:** 10.3390/v15051035

**Published:** 2023-04-23

**Authors:** Shouzhi Yu, Yangyang Wei, Hongyang Liang, Wenheng Ji, Zhen Chang, Siman Xie, Yichuan Wang, Wanli Li, Yingwei Liu, Hao Wu, Jie Li, Hui Wang, Xiaoming Yang

**Affiliations:** 1 Beijing Institute of Biological Products Company Limited, Beijing 100176, China; yushouzhi@sinopharm.com (S.Y.); yangyangwei3391@163.com (Y.W.); ayangyang@163.com (H.L.); 18298466258@163.com (W.J.); 13401114174@163.com (Z.C.); xsiman2017@163.com (S.X.); w250658055@163.com (Y.W.); lolired0704@163.com (W.L.); lywhing@126.com (Y.L.); wuhaobio@163.com (H.W.); lijie10201996@163.com (J.L.); 2 China National Biotec Group Company Limited, Beijing 100024, China

## Error in Table 3

In the original publication [1], there was a mistake in Table 3. ELISA analysis of protein and antigen concentrations of different inactivated virus samples as published. In the table, data in the second column (protein concentration) and the third column (antigen concentration) are raw data, and data in the fourth column were calculated from data in the third column divided by data in the second column. In the original publication, data in the fourth column were wrongly entered in. The corrected Table 3 appears below. The authors state that the scientific conclusions are unaffected. This correction was approved by the Academic Editor. The original publication has also been updated.

## Text Correction

With regard to the correction of Table 3, **a correction has been made to** *4. Discussion, Paragraph 4*:

**The results showed that formaldehyde treatment reduced the homogeneity of virus particles. Although there were no significant differences in the negative staining or circular dichroism spectra, the treament’s performance in terms of size exclusion chromatography became worse, and its UV absorption curve became less sharp. An examination of the S protein showed that the viral S protein after formaldehyde treatment displayed dispersive bands and cross-linking upon SDS-PAGE. Compared with the 20–24 h BPL treatment, the S antigen content decreased by more than twice. After BPL treatment, the virus particles remained relatively homogenous, and the S protein was still clear. These results confirm the chemical properties of formaldehyde, which can cause viral protein cross-linking and denaturation and damage viral surface antigens. Therefore, although formaldehyde does not destroy the morphology or secondary structure of the virus, compared with BPL, formaldehyde is not a suitable SARS-CoV-2 inactivator.**

## Figures and Tables

**Table 3:** ELISA analysis of protein and antigen concentrations of different inactivated virus samples.

Sample	Protein Concentration (μg/mL)	Antigen Concentration (U/mL)	Ratio (U/μg)
Formaldehyde-inactivated	332.4	267.9	0.81
Formaldehyde + BPL-inactivated	211.33	224.57	1.06
BPL-inactivated	327.56	623.38	1.90
BPL + BPL-inactivated	308.19	372.56	1.21
