# Supplementary material for: Comparison of Physical and Biochemical Characterizations of SARS-CoV-2 Inactivated by Different Treatments
Source: Viruses. 2022 Aug 31;14(9):1938. doi: 10.3390/v14091938 (PMC9503440; doi:10.3390/v14091938)
Supplement: Supplementary file 1 [file viruses-14-01938-s001.zip › viruses-1889480-supplementary.pdf]

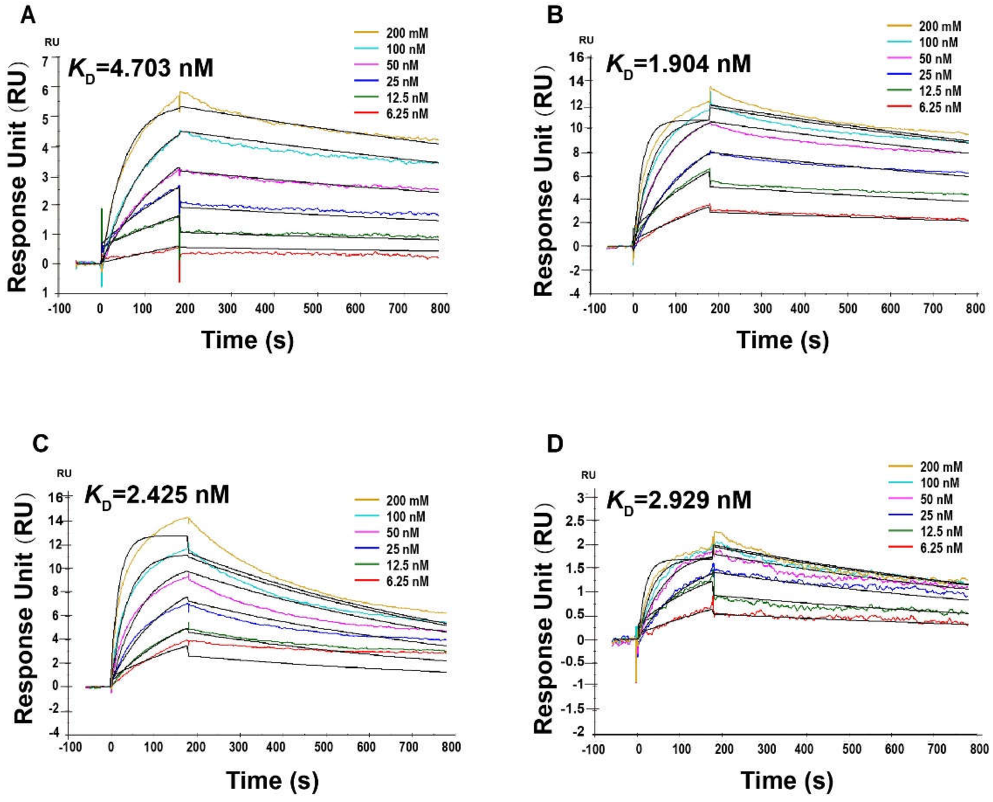

Figure S1 SPR analysis of different inactivated virus samples to SARS-CoV-2 Spike chimeric monoclonal antibody D002. (A) Formaldehyde-inactivated virus samples. (B) Formaldehyde+BPL-inactivated virus samples. (3) BPL-inactivated virus samples. (4) BPL+BPL-inactivated virus samples.
